# Austrian Syndrome in an Immunocompromised Patient—A Case Report

**DOI:** 10.3389/fmed.2020.00142

**Published:** 2020-04-21

**Authors:** Carlos E. Arias-Morales, Shorabh Sharma, Marjorie M. Flores-Chang, Razia Rehmani, Venkata Sandeep Koripalli, Michelle Dahdouh

**Affiliations:** ^1^Department of Internal Medicine, St. Barnabas Hospital Bronx, Bronx, NY, United States; ^2^School of Medicine, University of El Salvador, San Salvador, El Salvador; ^3^Department of Radiology, St. Barnabas Hospital Bronx, Bronx, NY, United States; ^4^Department of Medicine, Creighton University, Omaha, NE, United States; ^5^Division of Infectious Diseases, Department of Internal Medicine, St. Barnabas Hospital Bronx, Bronx, NY, United States

**Keywords:** *Streptococcus pneumoniae*, meningitis, endocarditis, pneumonia, invasive pneumococcal disease

## Abstract

Austrian syndrome consists of a triad of endocarditis, meningitis, and pneumonia caused by *Streptococcus pneumoniae*. With the arrival of many antibiotic therapies, the disease remains rare, however, it can be overlooked due to the lack of awareness. We present a case of Austrian syndrome in an immunocompromised patient complicated by multiorgan failure.

## Introduction

Austrian syndrome consists of a triad of endocarditis, meningitis, and pneumonia caused by *Streptococcus pneumoniae* (1). With the arrival of many antibiotic therapies, the disease remains rare, however, it can be overlooked due to the lack of awareness. Mortality in invasive pneumococcal disease appears to be increased in immunocompromised patients, especially HIV infected patients in whom the increase in mortality may be explained by the impaired humoral immunity that is needed to combat infections by encapsulated organisms, in this case, *S. pneumoniae* (2). To the best of our knowledge, there are ~ less than 60 cases reported in the medical literature. We present a case of Austrian syndrome in an immunocompromised patient complicated by multiorgan failure.

## Case Presentation

A 49-year-old female was brought to the emergency department due to altered mental status and generalized pain. The patient had a history of HIV on highly active antiretroviral therapy (HAART) with elvitegravir, cobicistat, emtricitabine, and tenofovir alafenamide (Genvoya®) and abacavir (Ziagen®), hepatitis C (treated), hypertension, diabetes mellitus type 2, asthma, active smoker, polysubstance use disorder including cocaine, marijuana, and alcohol. Allergy to penicillin manifested as rash and lip swelling was reported. On presentation, the patient was hypoxemic and lethargic for which she required intubation for airway protection. Exam was remarkable for tachycardia but with a regular rhythm, no murmurs were auscultated, lungs were clear, abdominal exam was benign, neurological exam prior to intubation only significant for lethargy, no neck stiffness or other meningeal signs were present. No further neurological exam was performed due to the patient being intubated.

### Investigations

**Figure 1:**
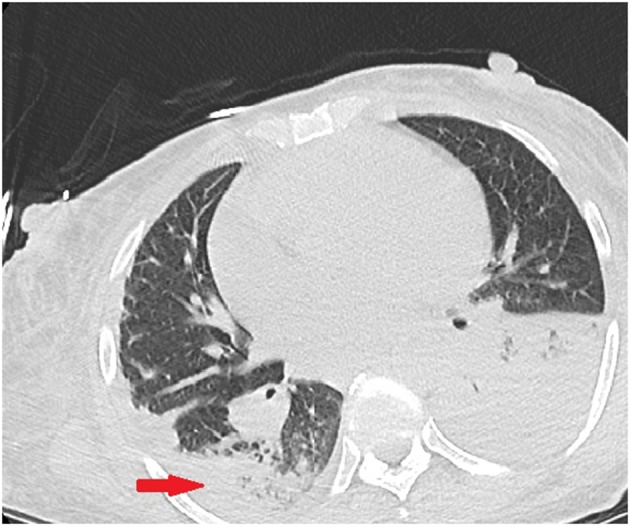
Initial CT chest without contrast demonstrating left lower lobe infiltrate and pleural effusion. Right lower lobe showing patchy reticulonodular interstitial lung disease.

Initial laboratory investigations showed a white blood count (WBC) of 22.8 × 10^3^/uL (reference range 4.0–10.0 10^3^/uL) with 87% neutrophils, serum creatinine of 4.3 mg/dL (reference range 0.6–1.2 mg/dL), blood urea nitrogen (BUN) of 73 (reference range 8–23 mg/dL), lactic acid of 8.8 (reference range: 0.0–2.2 mmol/L), CD4 count of 250 uL (reference range 359–1,519 uL), mild transaminitis. Blood cultures collected before the initiation of antibiotic therapy grew *Streptococcus pneumoniae* sensitive to ceftriaxone and penicillin. Sputum cultures grew *S. pneumoniae*. CT Chest without contrast reported diffuse interstitial lung disease with left lower lobe consolidation consistent with an infiltrate or consolidative atelectasis (Figure 1).

**Figure 2:**
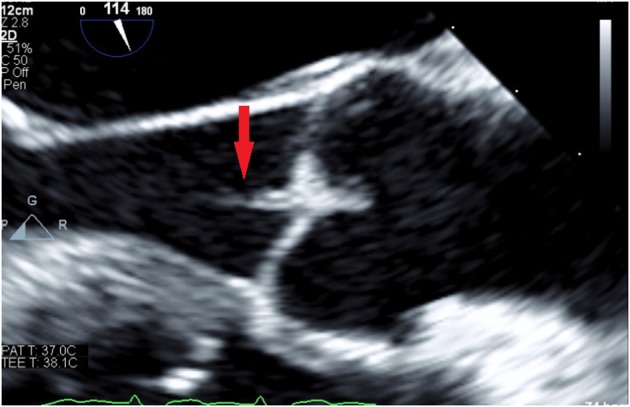
Transesophageal echocardiogram demonstrating small aortic valve vegetation (red arrow).

Transthoracic echocardiogram (TTE) was negative for vegetations. However, transesophageal echocardiogram (TEE) reported a small aortic valve vegetation, mild mitral and tricuspid valve regurgitation with a normal ejection fraction (Figure 2). The patient was started on intravenous vancomycin 15–20 mg/kg every 12 h and then changed to moxifloxacin 400 mg IV every 24 h (penicillin allergy and positive penetrance to central nervous system) and metronidazole 500 mg IV every 8 h based on final sensitivities. Atovaquone 750 mg via nasogastric tube twice a day was also started for the concern of PCP pneumonia and the presence of acute renal failure. The clinical team decided not to perform lumbar puncture due to the critical status of the patient and concerns for further complications such as bleeding. However, her mental status significantly improved a few days after initiation of antibiotic therapy with high nervous system penetrance. Additionally, the patient was started on renal replacement therapy with intermittent hemodialysis for acute kidney injury. Patient was weaned off mechanical ventilation, repeated blood cultures were negative, kidney injury resolved and the patient was successfully discharged from the hospital to a short term rehab (STR) facility. The patient was lost to follow up after STR.

## Informed Consent Statement

Verbal informed consent was obtained from the patient for publication of this report. All attempts were made to obtain written informed consent from the patient or patient's relative without success. The case report was submitted to the institutional review board at St. Barnabas Hospital and obtained approval and waived the requirement to obtain written informed consent for the publication of the case report, as this work does not contain any patient identifying information. IRB approval letter is available upon request.

## Discussion

Extracellular gram positive bacterial pathogens such as Streptococcus pneumoniae are highly invasive microorganisms that can cause severe and sometimes fatal, systemic infection (3). Morbidity and mortality rates due to pneumococcal disease remain significant, especially when complicated by endocarditis (4). Austrian syndrome consists of a triad of endocarditis, meningitis and pneumonia caused by S. pneumoniae. The entity was first described by Osler in 1881, however, the first published case was by Austrian in 1967 (1).

Several risk factors have been identified that contribute to invasive pneumococcal infections, such as debilitated patients (chronic alcoholism, diabetes mellitus, chronic kidney disease (CKD), liver and pulmonary disease, asplenism, immunosuppressed patients) (5). In the present case, our patient had several risk factors including a history of alcoholism, diabetes mellitus, CKD, and HIV. In a study by Gonzalez-Juanatey et al. HIV was identified in 6% of the patients. In patients with Austrian's syndrome, the aortic valve is involved in 75% of patients and valve replacement is required in 66% of patients (6). Our index patient, completed 6 weeks of antibiotics and did not require valvular replacement.

Invasive pneumococcal infection causes bacteremia and meningitis. Confirmation of *S. pneumoniae* isolation by a positive blood culture or cerebrospinal analysis (CSF) is required. Though we were initially unable to perform a lumbar puncture due to increased risk for bleeding and development of further complications, it was later deemed unnecessary, as the patient improved clinically with antibiotic therapy.

Since the introduction of antibiotics, the incidence of invasive pneumococcal infections has decreased, occurring in about 3% of the total cases of endocarditis, compared to 10–15% in the pre-antibiotic era (6, 7). Cardiac involvement is usually challenging, given that patients most of the times lack the classical features of infective endocarditis (8). TTE is known to be less sensitive than TEE for the diagnosis of infective endocarditis, as demonstrated in our case with a negative TTE but a positive TEE. Pneumococcal endocarditis is aggressive and rapidly progressive with a high potential for valvular destruction that translates into a higher mortality (9). Our patient not only had aortic valve vegetations, but also tricuspid and mitral valve dysfunction.

Limitations of treatment may include penicillin resistance in immunocompromised patients (HIV, malignancy, autoimmune disorders, transplant patients, chemotherapy, or patient who received antibiotics in the previous 3 months of pneumococcal infection). Vancomycin and ceftriaxone are recommended for initial empiric antibiotic therapy pending culture sensitivities, rifampin was added in age of pneumococcal resistance. Vancomycin with or without moxifloxacin is the best alternative choice in patients with a history of type I allergy to beta-lactam antibiotics who are not candidates for desensitization, as evidenced in our case. Adjunctive dexamethasone is recommended in documented pneumococcal meningitis. Monotherapy for endocarditis with penicillin, ceftriaxone or vancomycin once sensitivity is known is recommended and treatment duration is 4–6 weeks.

Additionally, pneumococcal serotypes 1, 3, and are more commonly known to cause invasive disease, but are not necessarily associated with worse outcomes (10).

With the arrival of many antibiotic therapies as well as pneumococcal vaccination, the disease remains rare, however, it can be overlooked due to the lack of awareness. Our case highlights the importance of obtaining timely blood cultures, and to consider invasive streptococcus pneumoniae infection in patients with suspected endocarditis and pneumonia with meningeal signs. To the best of our knowledge, there are ~ less than 60 cases reported in the medical literature.

## Conclusions

This case reports adds to the pool of literature describing this rare and high mortality disease, with the intention of creating awareness amongst physicians to emphasize the importance of pursuing an early diagnosis and identifying the appropriate treatments in order to reduce complications, as well as, create awareness of the importance of pneumococcal vaccination in vulnerable and the general population.

## Author Contributions

CA-M was the patient's physician, collected the data for case presentation, reviewed the literature and contributed to manuscript drafting. SS reviewed the literature and contributed to manuscript drafting. MF-C contributed with literature review and manuscript editing. RR was the patient's radiologist and contributed with figure description and manuscript drafting. MD performed the infectious diseases consultation, reviewed the literature, and drafted the manuscript. VK contributed with literature review and final manuscript drafting. All authors issued final approval for the version to be submitted.

## Conflict of Interest

The authors declare that the research was conducted in the absence of any commercial or financial relationships that could be construed as a potential conflict of interest.
